# Combined proteomic and transcriptomic analysis of the antimicrobial mechanism of tannic acid against *Staphylococcus aureus*

**DOI:** 10.3389/fphar.2023.1178177

**Published:** 2023-08-16

**Authors:** Jing Wang, Zhicun Sheng, Yunying Liu, Xiaolan Chen, Shuaibing Wang, Haifeng Yang

**Affiliations:** ^1^ Jiangsu Agri-Animal Husbandry Vocational College, Taizhou, Jiangsu Province, China; ^2^ Zhongchong Sino Biotech Taizhou Co., Ltd., Taizhou, Jiangsu Province, China

**Keywords:** *Staphylococcus aureus*, tannic acid, proteome, transcriptome, antimicrobial mechanism

## Abstract

*Staphylococcus aureus* is a zoonotic opportunistic pathogen that represents a significant threat to public health. Previous studies have shown that tannic acid (TA) has an inhibitory effect on a variety of bacteria. In this study, the proteome and transcriptome of *S. aureus* were analyzed to comprehensively assess changes in genes and proteins induced by TA. Initial observations of morphological changes revealed that TA damaged the integrity of the cell membrane. Next, proteomic and genetic analyses showed that exposure to TA altered the expression levels of 651 differentially expressed proteins (DEPs, 283 upregulated and 368 downregulated) and 503 differentially expressed genes (DEGs, 191 upregulated and 312 downregulated). Analysis of the identified DEPs and DEGs suggested that TA damages the integrity of the cell envelope by decreasing the expression and protein abundance of enzymes involved in the synthesis of peptidoglycans, teichoic acids and fatty acids, such as *murB, murQ*, *murG, fmhX* and *tagA*. After treatment with TA, the assembly of ribosomes in *S. aureus* was severely impaired by significant reductions in available ribosome components, and thus protein synthesis was hindered. The levels of genes and proteins associated with amino acids and purine synthesis were remarkably decreased, which further reduced bacterial viability. In addition, ABC transporters, which are involved in amino acid and ion transport, were also badly affected. Our results reveal the molecular mechanisms underlying the effects of TA on *S. aureus* and provide a theoretical basis for the application of TA as an antibacterial chemotherapeutic agent.

## 1 Introduction

*Staphylococcus aureus* is a widely distributed zoonotic opportunistic pathogen, as well as one of the most common food-borne diseases (Khanal et al., 2022). *S. aureus* usually causes mild skin or wound infections, and in severe cases, systemic infections such as sepsis (Woolhouse et al., 2015). *S. aureus* infection not only poses a threat to human health, but also causes severe economic losses to the animal husbandry industry. The most significant challenge facing the development of effective treatments for *S. aureus* infection is the rapid development of antibiotic resistance (Wencewicz, 2019). As a result of this characteristic of *S. aureus*, the development of new antimicrobial drugs lags far behind the emergence of resistant bacteria (Hutchings et al., 2019). Therefore, new antibacterial agents with activity against *S. aureus* are always urgently needed. However, in the past 50 years, only two synthetic antibiotics, fluoroquinolones and oxazolidinones, have been developed successfully (Lewis, 2013).

Given the successful development of drugs from natural products, such as lovastatin and reserpine, increasing attention has been paid to the therapeutic potential of natural products in recent years. Among such natural products, tannins are a group of polyphenols that are widely present in many types of trees and higher plants, such as green tea, coffee, and fruit species (Chung et al., 1998a). Over thousands of years, diverse tannin-containing plant species in China and across Asia, such as *Galla chinensis*, have been used as astringents to treat diarrhea and hemorrhage (Djakpo and Yao, 2010), as well as administered as anticarcinogens (Zhang et al., 2015; Gao et al., 2018) and antimicrobial agents (Chung et al., 1998a).

**FIGURE 1:**
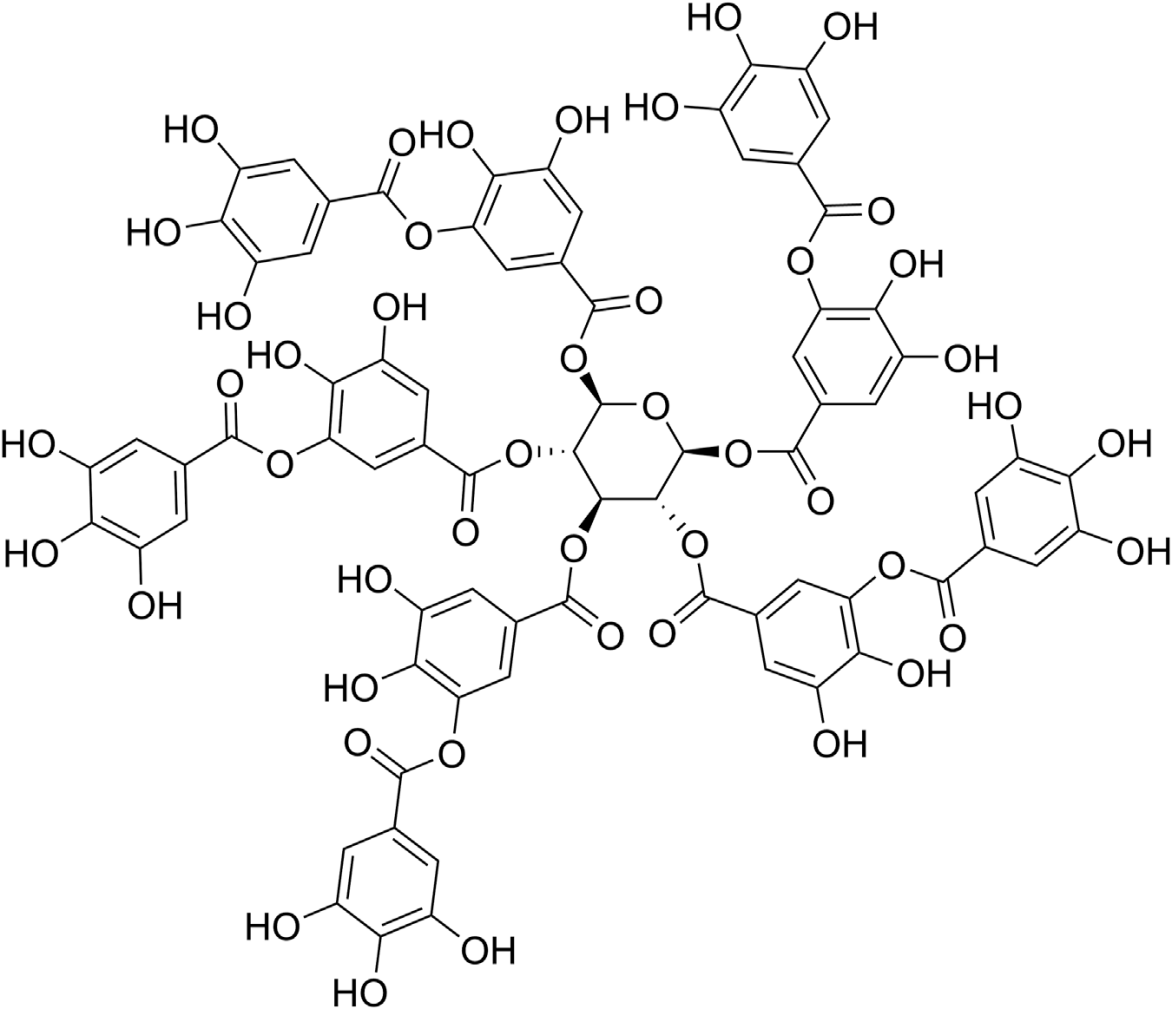
The chemical structure of TA.

Tannic acid (TA, Figure 1) is the simplest hydrolysable tannin and is approved as a weakly acidic food additive by the US Food and Drug Administration (FDA) (Guo et al., 2021). It has attracted much attention in recent years due to its extensive physiological activities, which include antioxidant, antitumoral, antimicrobial and anti-inflammatory actions, in addition to its ability to interact with proteins, as well as its potential in materials science and engineering applications (Guo et al., 2021). TA is composed of a core glucose molecule connected to 10 galloyl groups by aliphatic ester bonds (Guo et al., 2021). A clinical study has shown that Cesinex^®^, a TA-based medical food, is effective against broad-spectrum diarrhea and has a good safety profile (Ren et al., 2012).

Previous work revealed that TA has an inhibitory effect on a variety of bacteria, including *S. aureus, Salmonella,* and *Lactobacillus* (Taguri et al., 2004). More than that, TA could enhance the inhibitory effect of antibiotics such as erythromycin on drug-resistant *S. aureus* through suppression the drug efflux pump (Tintino et al., 2016; Tintino et al., 2017). Chung et al. suggested that TA inhibited bacterial growth by chelating ions from the culture medium (Chung et al., 1998b). It has also been reported that TA inhibits bacterial growth by inhibiting fatty acid synthesis through suppression of the activity of β-ketoacyl-ACP reductase (Wu et al., 2010). Other research has indicated that TA binds directly to peptidoglycans (PGs) in the bacterial cell wall, interfering with cell wall integrity (Dong et al., 2018). However, the mechanisms underlying the inhibitory effect of TA on *S. aureus* have not been assessed comprehensively. Therefore, the present study combined transcriptome and proteome data to analyze the molecular mechanisms of TA in *S. aureus* and identify potential targets for the development of new therapeutic agents from natural sources.

## 2 Materials and methods

### 2.1 Bacterial strains and chemicals

The *S. aureus* strain ATCC 6538 was purchased from the American Type Culture Collection. The clinical strains used in this study were isolated from milk. All strains were maintained in a Microbank (Huankai Microbial, China) at −80°C until use. Tannic acid (TA) was purchased from Chengdu Herbpurify Co., LTD (Chengdu, China, CAS No.: 1401-55-4). TA was stored at −20°C and pre-dissolved with ultrapure DMSO before use.

### 2.2 Determination of the minimum inhibitory concentration (MIC) of TA and growth curves

The MIC of TA against *S. aureus* was determined by the Broth Microdilution Method (Rajagopal and Walker, 2017). The cell concentration was adjusted to approximately 5 × 10^5^ cells/mL using MHB medium. The MIC was the minimum concentration of the test drug that inhibited visible growth of the bacteria after the plates were incubated at 37°C for 16 h. All experiments were performed with three replicates. To determine the growth curve of *S. aureus*, 0.5 mL of fresh *S. aureus* (adjusted to OD_600_ = 1.0) was added to 50 mL of sterile TSB broth with or without the 1/2 MIC of TA. The absorbance value at 600 nm (OD_600_) was measured every 2 h for 24 h using a Multiskan GO instrument (Thermo Scientific, United States).

### 2.3 Scanning electron microscopy observations

To observe the morphological effect of TA on *S. aureus*, scanning electron microscopy (SEM) imaging was performed according to previously described methods (Zhou et al., 2022). The concentration of fresh bacteria was adjusted to OD_600_ = 1.0 using sterilized MHB medium. The cell suspension was inoculated into sterilized MHB with or without 1/2 MIC TA at a rate of 1:100. After incubation at 37°C for 5 h with shaking at 170 rpm, cells were collected by centrifugation and washed three times with sterilized 0.1 M phosphate-buffered saline (PBS). Bacterial pellets were gently resuspended, fixed with 2.5% glutaraldehyde for 4 h, and then washed three times with PBS. The samples were fixed with 1% osmium tetraoxide at room temperature for 1 h. Subsequently, the samples were dehydrated with gradually increasing concentrations of ethyl alcohol (30, 50, 70, 85, 95, and 100%). The samples were freeze-dried overnight and sputter-coated with gold prior to observation using a Regulus-8100 system (Hitachi, Japan).

### 2.4 Confocal microscopy assay

The cell membrane integrity of *S. aureus* was observed by confocal fluorescence microscopy (Acebron-Garcia-de-Eulate et al., 2022). The fresh bacteria were washed three times with sterilized PBS and adjusted to obtain a bacterial suspension with a density of approximately 1.5 × 10^6^ cfu, after which the samples were incubated with the 1/2 MIC concentration of TA or lysostaphin (8 μg/mL) for 30 min. After incubation, bacterial cells were washed with PBS and then stained with 5 µM PI and 10 µM SYTO™ 9 Green Fluorescent Nucleic Acid Stain (Thermo Fisher Scientific, Waltham, United States). Finally, the images were analyzed and merged using LAS AF Lite software (Leica).

### 2.5 Sample preparation

The *S. aureus* ATCC 6538 solution (OD_600_ = 1.0) was inoculated into 100 mL MHB, and TA was added to reach the 1/2 MIC concentration (16 μg/mL). The control group was not treated with TA. Three independent cultures with or without TA were prepared for both transcriptome and proteome sequencing. The TA groups were designated as TA1, TA2, and TA3. The control groups were designated as C1, C2, and C3. Samples were collected by centrifugation at 5,000 rpm for 10 min after incubation with shaking at 37°C for 5 h. The samples were washed three times with PBS to remove residual medium and drugs. All samples were divided into two parts for proteome and transcriptome sequencing. The samples were stored at −80°C before RNA or protein extraction.

### 2.6 Combined transcriptome and proteome analysis

iTRAQ and transcriptome analysis were carried out at Shanghai Majorbio Biopharm Technology Co. Ltd. (Shanghai, China). The protocols for proteome, transcriptome and RNA-protein correlation analysis are described briefly here. For proteomics, 6 samples were extracted by protein extraction buffer (including 1% SDS, 200 mM DTT, 50 mM Tris-HCl and protease inhibitor, pH 8.8). The total protein abundance was quantitated by the BCA method. About 100 µg of each sample was added to triethylammonium bicarbonate buffer to achieve reduction alkylation, and trypsin was added for digestion. Furthermore, all samples were subjected to TMT labeling (Thermo Fisher No. 90111), C18 reverse phase liquid chromatography fractionation, and liquid chromatography coupled with tandem mass spectrometry (LC-MS/MS) (Q_Exactive HF-X, Thermo, USA). The *m*/*z* scan range of MS was 350–1,500. A protein was considered to be differentially expressed if its fold change was greater than 1.2-fold (*p* < 0.05) or less than 0.83-fold (*p* < 0.05) according to the comparison between the treatment and control groups using Student’s *t*-test.

For transcriptome analysis, total RNA from each sample was extracted using Trizol reagent (Invitrogen, Carlsbad, CA) according to the instructions from the manufacturer. The cDNA library was constructed using the Illumina TruSeq™ Stranded Total RNA Library Prep Kit after analysis to determine the quantity of total RNA. Transcriptomics sequencing was performed on an Illumina HiSeq platform. Data analyses were performed using DESeq2 software. Genes with P-adjust <0.05 and |log2 fold change| ≥ 1 were considered to be differentially expressed genes (DEGs).

### 2.7 Bioinformatics

The function and pathway enrichment analyses were performed on the identified DEGs and DEPs. The data were analyzed on the online Majorbio Cloud Platform (www.majorbio.com). Functional analysis was conducted using Goatools software with P-adjust <0.05 as the threshold, and the proteins and genes were classified into biological processes, molecular functions and cellular compartments. The DEGs and DEPs were further annotated using the KEGG database (http://www.genome.jp/kegg/). KEGG pathway enrichment analysis was further performed to determine the metabolic and signaling pathways enriched among the differentially expressed proteins and genes.

### 2.8 Quantitative determination of gene expression level

Quantitative real-time PCR (qRT-PCR) was conducted to confirm the differentially expressed proteins and genes from the proteome and transcriptome sequencing experiments. *S. aureus* ATCC 6538 was cultured under the same conditions used for the samples in the omics sequencing experiments. Total RNA was extracted from the TA and control groups using the TaKaRa MiniBEST Universal RNA Extraction Kit (TaKaRa, Dalian, China) according to the manufacturer’s protocol. 18 DEGs were selected for qRT-PCR analysis, and 16 S rRNA was used as the internal reference gene. qRT-PCR was performed by a two-step process using the PrimeScript™ RT Master Mix Kit and TB Green Premix Ex Taq TM II Kit (TaKaRa, Dalian, China). All genes were independently assayed three times. The relative quantitation of the mRNA expression level of each gene was calculated by the 2^−∆^^∆^ CT method. All of the primers and sequences are listed in Supplementary Table S1.

## 3 Results

### 3.1 Inhibitory activity of TA against *S. aureus*

**FIGURE 2:**
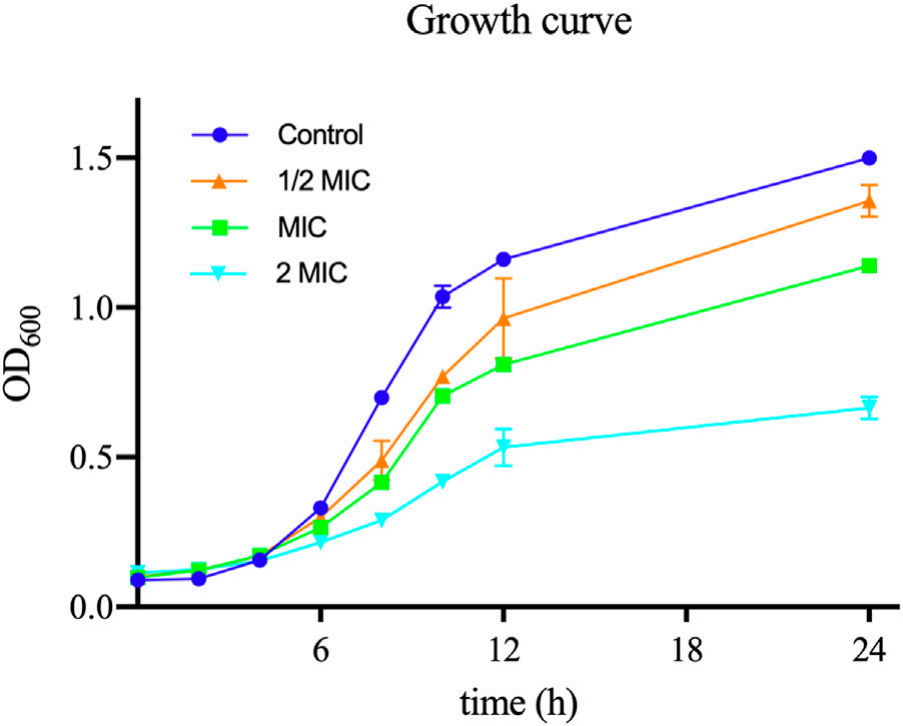
*S. aureus* growth curves during treatment with different concentrations of TA at 37°C.

In order to assess the antibacterial activity of TA, we measured the MIC of TA against 11 strains of *S. aureus*: the ATCC 6538 strain and 10 clinical strains. The MIC value of TA for ATCC 6538 was 32 μg/mL, whereas the MIC values of the selected clinical strains of *S. aureus* ranged from 32 μg/mL to 128 μg/mL. As shown in Figure 2, the growth rates of *S. aureus* planktonic cells exposed to different concentrations of TA were slower than that of the control group.

### 3.2 TA affected the membrane morphology and permeability of *S. aureus*

**FIGURE 3:**
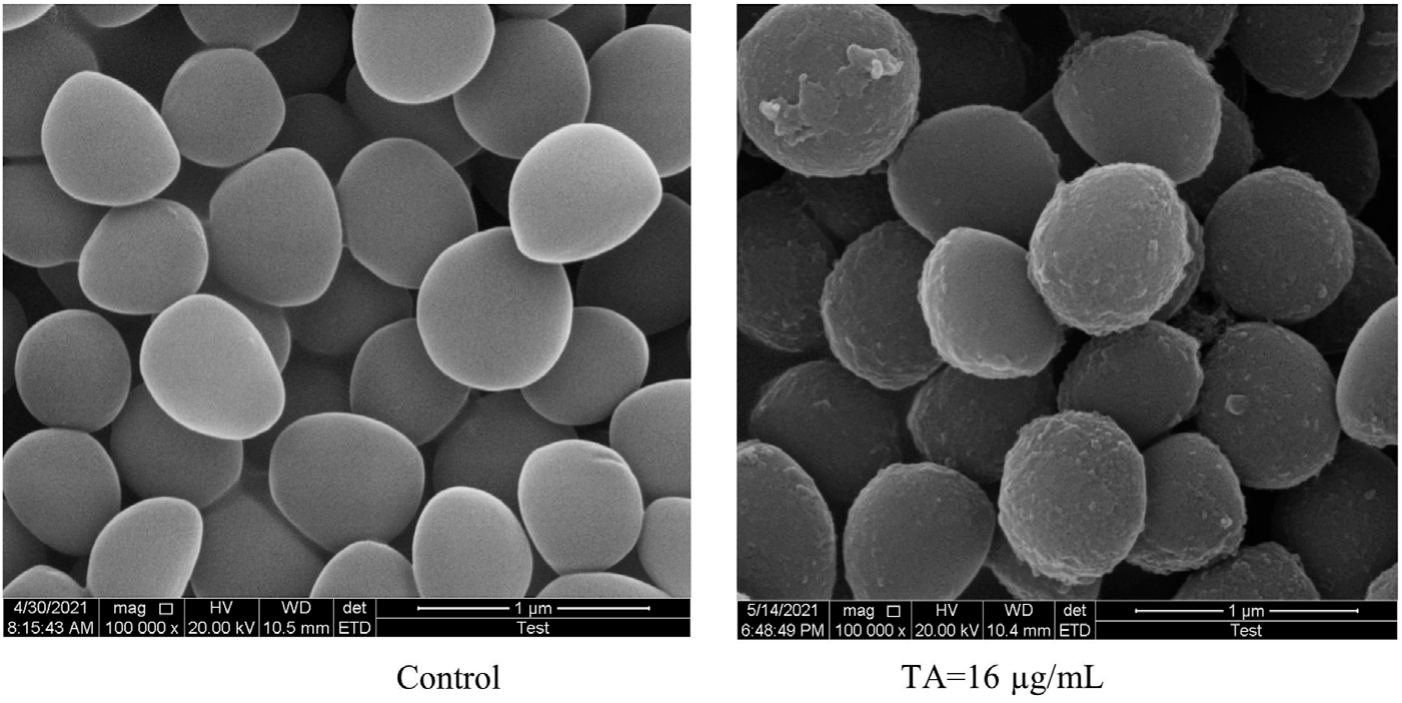
Scanning electron microscopy images showing the structure of *S. aureus* treated with 1/2 MIC TA for 6 h. Magnification: 1 000 000×.

**FIGURE 4:**
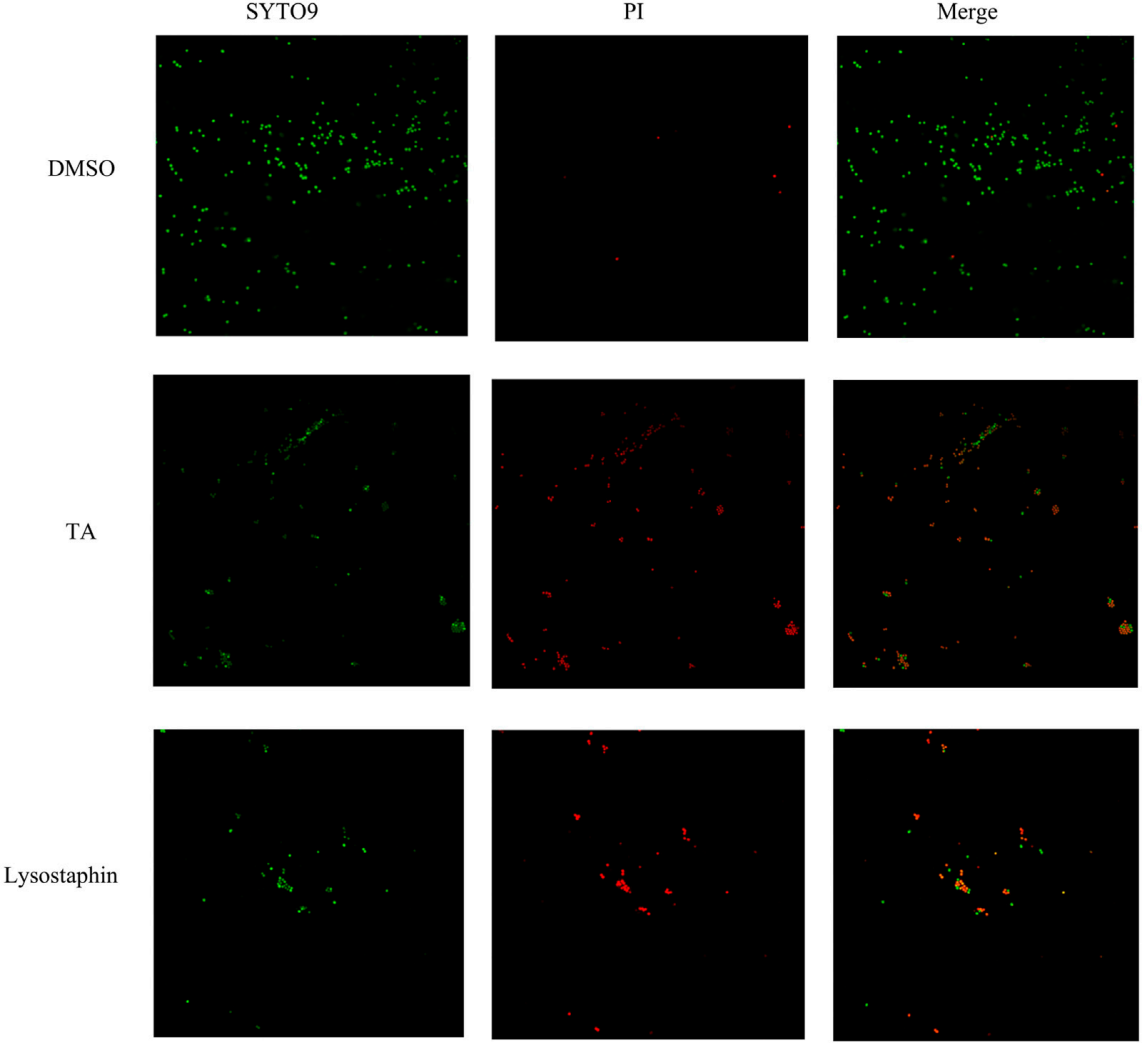
TA disrupted the bacterial membrane integrity of *S. aureus*.

Scanning electron microscopy (SEM) was utilized to observe the morphology of *S. aureus* treated with TA. The SEM images showed that the bacteria tended to form stacks rather than a single layer of cells. The cell walls of ATCC 6538 exhibited contraction and increased roughness after treatment with TA (Figure 3). As shown in Figure 4, confocal microscopy showed that treatment with 16 μg/mL TA (1/2 MIC) disrupted the integrity of the cell membrane of *S. aureus.* After incubation with TA for 30 min, the number of bacteria stained with green fluorescence by PI was significantly greater than that of the control group.

### 3.3 Proteomic analysis of *S. aureus* upon TA treatment

**FIGURE 5:**
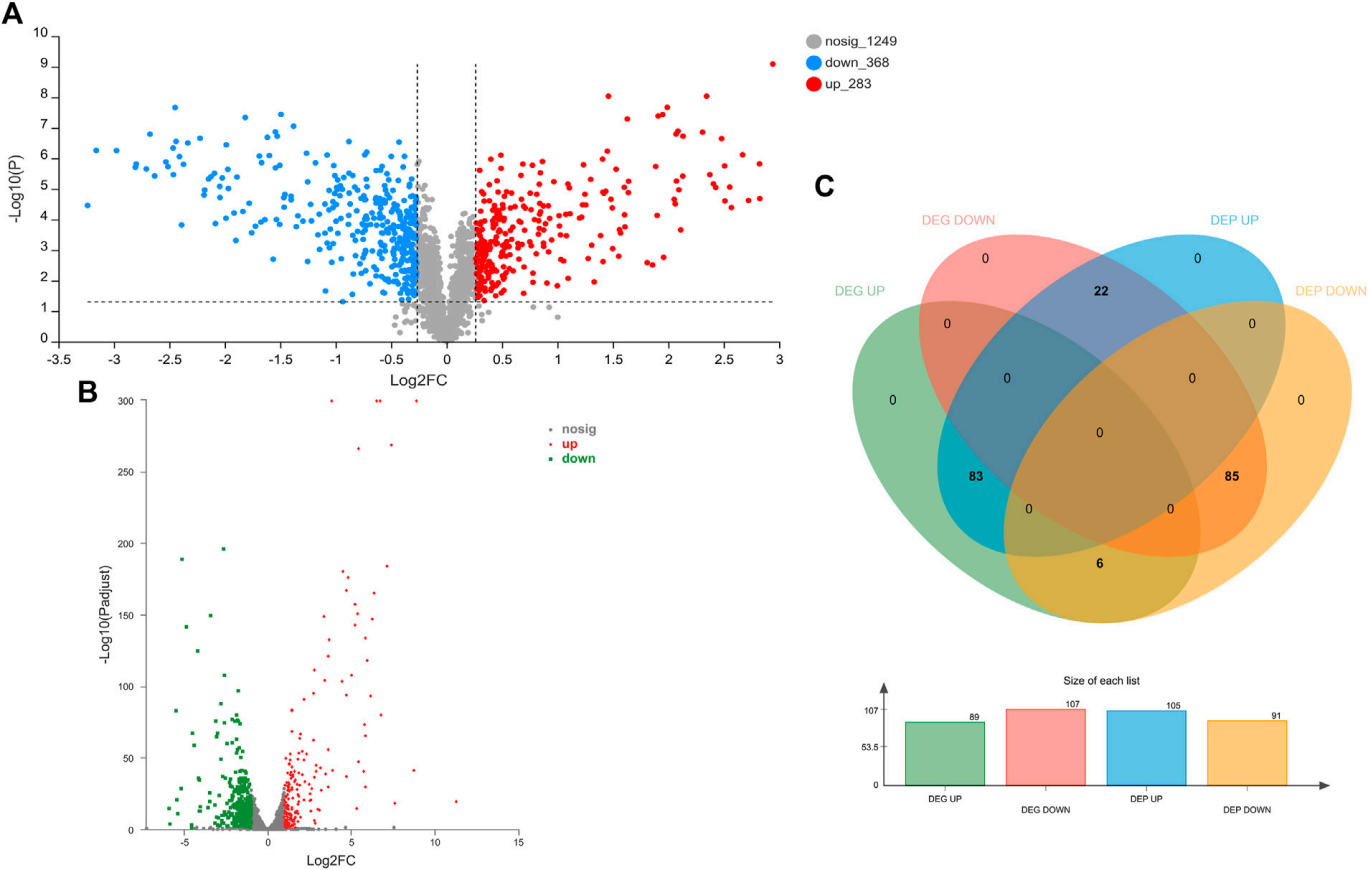
Proteomic and transcriptomic changes in *S. aureus* in response to the treatment with 1/2 MIC TA. **(A,B)** Volcano plots of the relative abundance of proteins **(A)** or transcripts **(B)**; **(C)** Venn diagram of DEGs and DEPs in the TA group *vs.* control group.

The influence of TA treatment on *S. aureus* at the protein level was evaluated by the iTRAQ method. A total of 1900 proteins were identified in *S. aureus* in this study. Differentially expressed proteins (DEPs) were defined as follows: fold change (FC) > 1.20 or <0.87 and *p* < 0.05. The comparison of control and TA-treated *S. aureus* revealed 651 significant DEPs (283 upregulated proteins and 368 downregulated proteins). Figure 5 shows volcano plots of FC values against *p*-values (two-tailed Student’s test), along with the number of differentially expressed proteins and genes.

**FIGURE 6:**
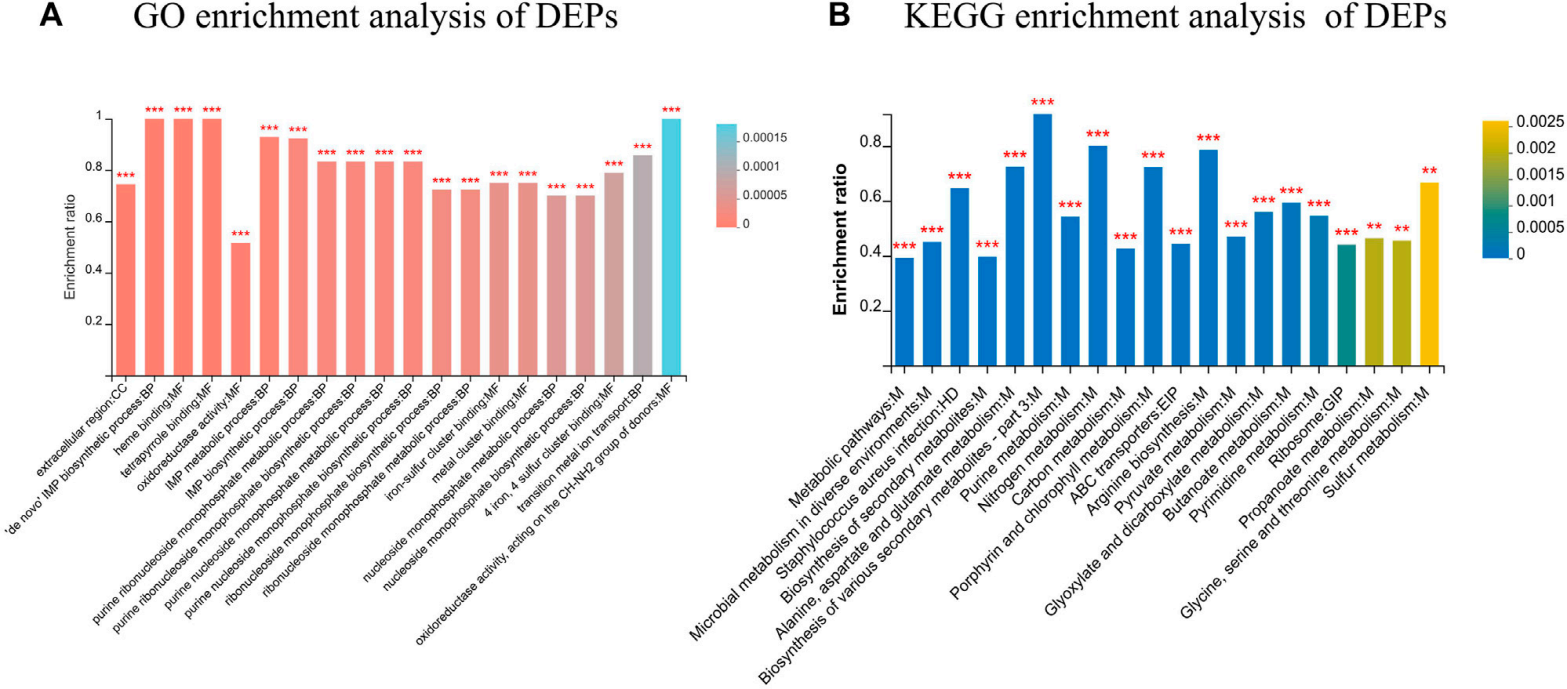
Functional classification of DEPs. **(A)** GO enrichment analysis of DEPs; **(B)** KEGG enrichment analysis of DEPs. *FDR <0.05, **FDR <0.01, ***FDR <0.001.

GO annotation of the set of 651 DEPs revealed 25 functional terms, including 10 biological processes (BP), 2 cellular components (CC) and 13 molecular functions (MF). GO functional enrichment analysis of the DEPs showed that the 20 most significantly enriched GO terms were mainly BPs and MFs, including oxidoreductase activity (GO: 0016491), purine ribonucleoside monophosphate biosynthetic process (GO:0009168), and IMP metabolic process (GO: 0046040) (*p* < 0.001). Moreover, KEGG pathway analysis revealed components of 93 KEGG pathways among the 651 DEPs, the majority of which were associated with global and overview maps. According to the KEGG enrichment analysis (Figure 6), DEPs were significantly enriched in metabolic pathways, microbial metabolism in diverse environment, *Staphylococcus aureus* infection, and biosynthesis of secondary metabolites, among other terms.

### 3.4 Transcriptomic analysis of *S. aureus* upon TA treatment

The effects of TA treatment on the *S. aureus* transcriptome were determined by RNA-seq experiments. A total of 2,590 mRNA were identified in this study. Differentially expressed genes (DEGs) were defined as follows: corrected *p*-value <0.05 and |log2 FC| ≥ 1. The comparison of control and TA-treated *S. aureus* revealed 503 DEGs (191 upregulated genes and 312 downregulated genes) (Figure 5B).

**FIGURE 7:**
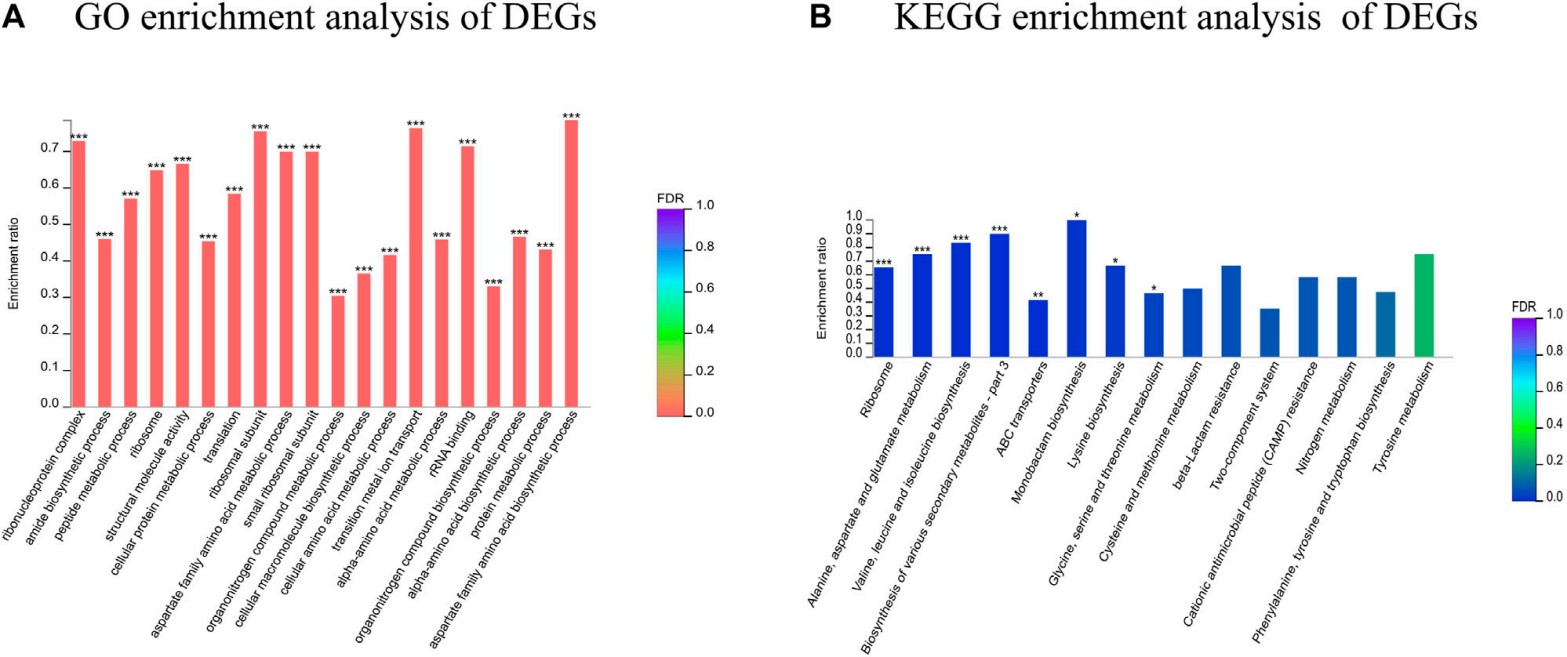
Functional classification of DEGs. **(A)** GO enrichment analysis of DEGs; **(B)** KEGG enrichment analysis of DEGs. *FDR<0.05, **FDR<0.01, ***FDR<0.001.

GO annotation analysis of the set of 503 DEGs revealed 488 functional terms, including 269 MFs, 28 CCs and 191 BPs. GO functional enrichment analysis of the DEGs showed that most of the top enriched terms were BPs and MFs, including ribonucleoprotein complex (GO: 1990904), amide biosynthetic process (GO: 0043604) and peptide metabolic process (GO:0006518) (Figure 7). KEGG pathway annotation and enrichment analysis indicated that the DEGs were enriched in several pathways, including ribosome; metabolism of alanine, aspartate and glutamate; biosynthesis of valine, leucine and isoleucine; and ABC transporters, among others.

### 3.5 Correlation analysis of DEGs and DEPs

**FIGURE 8:**
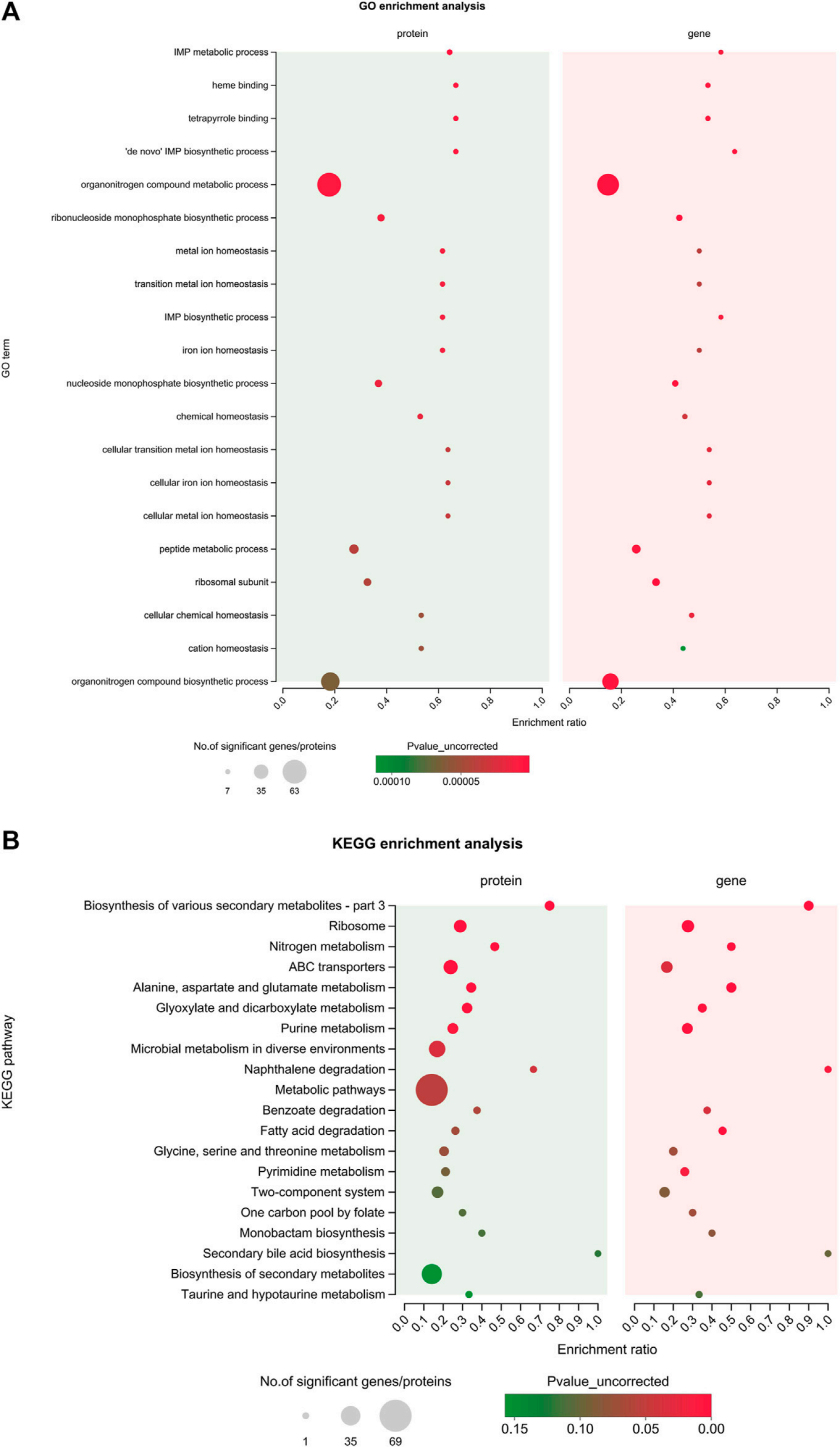
Integrated analysis of the proteomic and transcriptomic data. **(A)** The top 20 enriched GO terms of the integrated DEGs and DEPs; **(B)** The top 20 enriched KEGG pathways of the integrated DEGs and DEPs.

In order to explore the relationship between mRNA and protein expression profiles, an integrated analysis of the proteome and transcriptome data was performed on the online Majorbio Cloud Platform (www.majorbio.com). The results showed that all of the quantified proteins corresponded to those inferred by the RNA-seq data. The analyses revealed that 196 differentially significant proteins were detected by both transcriptome and proteome sequencing. The proteome and transcriptome analyses revealed that 99 proteins and their corresponding genes were upregulated, whereas 116 were downregulated (Figure 5C). Twenty-two proteins were found to be upregulated by proteome sequencing, but transcriptome analysis revealed that their genes were downregulated, while eight proteins showed the opposite relationship (downregulated DEP and upregulated DEG). Further, the sets of DEPs and DEGs were subjected to functional enrichment analysis. The top 20 significantly enriched GO terms and KEGG pathway for the DEGs and DEPs are shown in Figure 8. The IMP metabolic process (GO: 0046040), heme binding (GO: 0020037), and tetrapyrrole binding (GO: 0046906) GO terms were very significantly enriched (FDR <0.001) in the DEGs and DEPs, while the biosynthesis of various secondary metabolites, ribosome, nitrogen metabolism and ABC transporters KEGG pathways were also enriched significantly (FDR <0.05). The key DEGs and DEPs in *S. aureus* exposed to TA are listed in Table 1.

**TABLE 1:** Key DEGs and DEPs in *S. aureus* exposed to TA.

Accession	Gene name	Protein ID	Description	Log2 FC	
				Gene	Protein
Cell envelope					
B4602_RS03795	*murB*	WP_000608440.1	UDP-N-acetylenolpyruvylglucosamine reductase	−0.21	−0.36
B4602_RS07210	*murG*	WP_000160904.1	undecaprenyldiphospho-muramoylpentapeptide beta-N-acetylglucosaminyltransferase	−0.34	−0.38
B4602_RS00765	*murQ*	WP_042727603.1	N-acetylmuramic acid 6-phosphate etherase	0.42	−0.72
B4602_RS11895	*fmhX*	WP_000413866.1	Peptidoglycan interpeptide bridge formation enzyme	−0.47	−0.43
B4602_RS03225	*tagA*	WP_000215388.1	WecB/TagA/CpsF family glycosyltransferase	−0.21	−0.33
B4602_RS03245	-	WP_001241179.1	Glycosyltransferase family 2 protein	0.62	−0.29
B4602_RS08180	*accC1*	WP_000942744.1	Acetyl-CoA carboxylase biotin carboxylase subunit	−0.79	−1.16
B4602_RS08185	*BCCP*	WP_001019340.1	Acetyl-CoA carboxylase biotin carboxyl carrier protein subunit	−1.03	−0.88
B4602_RS05185	*purK*	WP_001010407.1	5-(carboxyamino)imidazole ribonucleotide synthase	−0.90	−2.44
B4602_RS05935	*carB*	WP_001126265.1	Carbamoyl-phosphate synthase large subunit	−2.13	−0.78
B4602_RS09915	-	WP_000544969.1	Type 1 glutamine amidotransferase	−0.16	−0.34
Ribosome and protein synthesis					
B4602_RS14385	*rpmH*	WP_000240855.1	50S ribosomal protein L34	−0.49	−3.24
B4602_RS11790	*rplP*	WP_000926310.1	50S ribosomal protein L16	−1.62	−0.48
B4602_RS11810	*rplB*	WP_000985472.1	50S ribosomal protein L2	−1.86	−0.96
B4602_RS11710	*rpmJ*	WP_000868342.1	50S ribosomal protein L36	−0.85	−2.7
B4602_RS06750	*rpsN*	WP_001085655.1	30S ribosomal protein S14	−2.56	−3.16
B4602_RS11660	*rpsI*	WP_001790547.1	30S ribosomal protein S9	−0.99	−1.25
B4602_RS01735	*rpsR*	WP_000897044.1	30S ribosomal protein S18	−1.62	−0.98
B4602_RS06280	*infB*	WP_000043635.1	Translation initiation factor IF-2	0.28	−0.43
B4602_RS08120	*rsfS*	WP_042727695.1	Ribosome silencing factor	0.5	0.39
B4602_RS03865	*raiA*	WP_079199722.1	Ribosome-associated translation inhibitor RaiA	0.3	0.77
B4602_RS00050	*serS*	WP_000884334.1	Serine--tRNA ligase	−2.72	−0.58
B4602_RS03545	*ybaK*	WP_001007235.1	Cys-tRNA (Pro) deacylase	−0.57	−0.37
B4602_RS13690	*QueH*	WP_000606207.1	Epoxyqueuosine reductase	−1.16	−0.73
Amino acids and purine biosynthesis					
B4602_RS05930	*carA*	WP_042727655.1	Carbamoyl-phosphate synthase small subunit	−2.23	−0.63
B4602_RS05935	*carB*	WP_001126265.1	Carbamoyl-phosphate synthase large subunit	−2.13	−0.79
B4602_RS06480	*glnA*	WP_001126603.1	Glutamine synthetase	−2.14	−0.42
B4602_RS06715	*thrB*	WP_000073182.1	Homoserine kinase	−2.93	−0.36
B4602_RS07310	*tdcB*	WP_000210820.1	Bifunctional threonine ammonia-lyase/L-serine ammonia-lyase	−2.78	−2.08
B4602_RS05210	*purF*	WP_000483720.1	Amidophosphoribosyltransferase	−1.16	−2.53
B4602_RS05200	*purQ*	WP_000666806.1	Phosphoribosylformylglycinamidine synthase I	−1.41	−2.46
B4602_RS05220	*purN*	WP_000238669.1	phosphoribosylglycinamide formyltransferase, partial	−1.33	−2.45
B4602_RS10010	*purB*	WP_000572878.1	Adenylosuccinate lyase	0.32	−1.02
B4602_RS00095	-	WP_000095328.1	Adenylosuccinate synthase	−1.47	−1.06
B4602_RS13495	*pruA*	WP_000259692.1	L-glutamate gamma-semialdehyde dehydrogenase	0.48	0.39
B4602_RS05360	*lpdA*	WP_000260117.1	Dihydrolipoamide dehydrogenase of pyruvate dehydrogenase complex	0.60	0.52
B4602_RS00640	-	WP_000290396.1	Aldehyde dehydrogenase family protein	0.35	0.52
ABC transporter					
B4602_RS12705	*hisM*	WP_000479576.1	Amino acid ABC transporter, permease protein	0.64	−0.36
B4602_RS02155	*gmpC*	WP_000825526.1	Dipeptide ABC transporter glycylmethionine-binding lipoprotein	0.40	−0.29
B4602_RS11480	-	WP_000989091.1	Branched-chain amino acids transporter, Periplasmic Binding Protein (PBP)-dependent ATP-Binding protein	2.47	1.00
B4602_RS13020	*oppF*	WP_000590512.1	Oligopeptide ABC transporter ATP-binding protein	1.81	0.53
B4602_RS13025	*oppD*	WP_000173875.1	Oligopeptide ABC transporter ATP-binding protein	1.83	0.85
B4602_RS12920	*opuBB*	WP_000398941.1	ABC-type proline/glycine betaine transport system	−0.38	−1.02
B4602_RS12925	*opuCC*	WP_000721551.1	ABC-type osmoregulatory transporter	−0.24	−0.98
B4602_RS12935	*opuBA*	WP_000948979.1	ABC-type proline/glycine betaine transport system	0.08	−1.53
B4602_RS03200	*psaA*	WP_000737654.1	Metal ABC transporter, substrate-binding protein	−4.90	−0.94
B4602_RS05555	*isdF*	WP_000594541.1	ABC-type Fe^3+^-siderophore transport system, permease component	5.78	1.64
B4602_RS00370	*sirA*	WP_001045111.1	Staphyloferrin B ABC transporter substrate-binding protein	6.73	2.48
B4602_RS03280	*fepC*	WP_001080809.1	ABC-type cobalamin/Fe3+-siderophores transporter, ATP-binding protein	2.74	1.30
B4602_RS11485	*fecCD*	WP_000974906.1	Iron ABC transporter, permease protein	3.03	0.87
B4602_RS11490	*fecB*	WP_001214661.1	Fe^3+^-citrate transport system, substrate-binding protein	4.70	1.99
B4602_RS13040	*cntA*	WP_001229083.1	Staphylopine-dependent metal ABC transporter, substrate-binding protein	1.81	1.26
B4602_RS03780	*ceuA*	WP_000754443.1	Siderophore ABC transporter, substrate-binding protein	4.51	2.13
B4602_RS00885	*ugpA*	WP_042727601.1	Sugar ABC transporter permease	−0.28	−0.64
B4602_RS00875	*ugpC*	WP_000818913.1	Sn-glycerol-3-phosphate ABC transporter, ATP-binding protein	−0.52	−0.42
B4602_RS13905	*lolD*	WP_000923760.1	ABC-type lipoprotein export system, ATP-binding protein	−0.84	−0.35
B4602_RS00520	*phnD*	WP_000787672.1	Phosphated ABC transporter, substrate-binding protein	−0.08	−0.58
B4602_RS00510	*phnE*	WP_001127863.1	Phosphonate ABC transporter, permease protein	−0.86	−0.40
Two-component system					
B4602_RS07600	*srrA*	WP_000064078.1	Two-component system sensor histidine kinase SrrA	−0.11	−0.50
B4602_RS07595	*srrB*	WP_000987769.1	Two-component system sensor histidine kinase SrrB	−0.35	−0.27
B4602_RS14300	*vraD*	WP_000154162.1	ABC transporter, ATP-binding protein, partial	−5.49	−1.14
B4602_RS14305	*vraE*	WP_000143652.1	Peptide resistance ABC transporter permease subunit	−5.19	−1.06
B4602_RS03355	*vraF*	WP_000985996.1	ABC transporter ATP-binding protein	−1.05	−0.29
B4602_RS00915	*uhpT*	WP_001008722.1	Hexose-6-phosphate: phosphate antiporter	−1.21	−0.53
B4602_RS01570	*glpT*	WP_001010111.1	Glycerol-3-phosphate transporter	−2.22	−0.29

### 3.6 qRT-PCR validation

**FIGURE 9:**
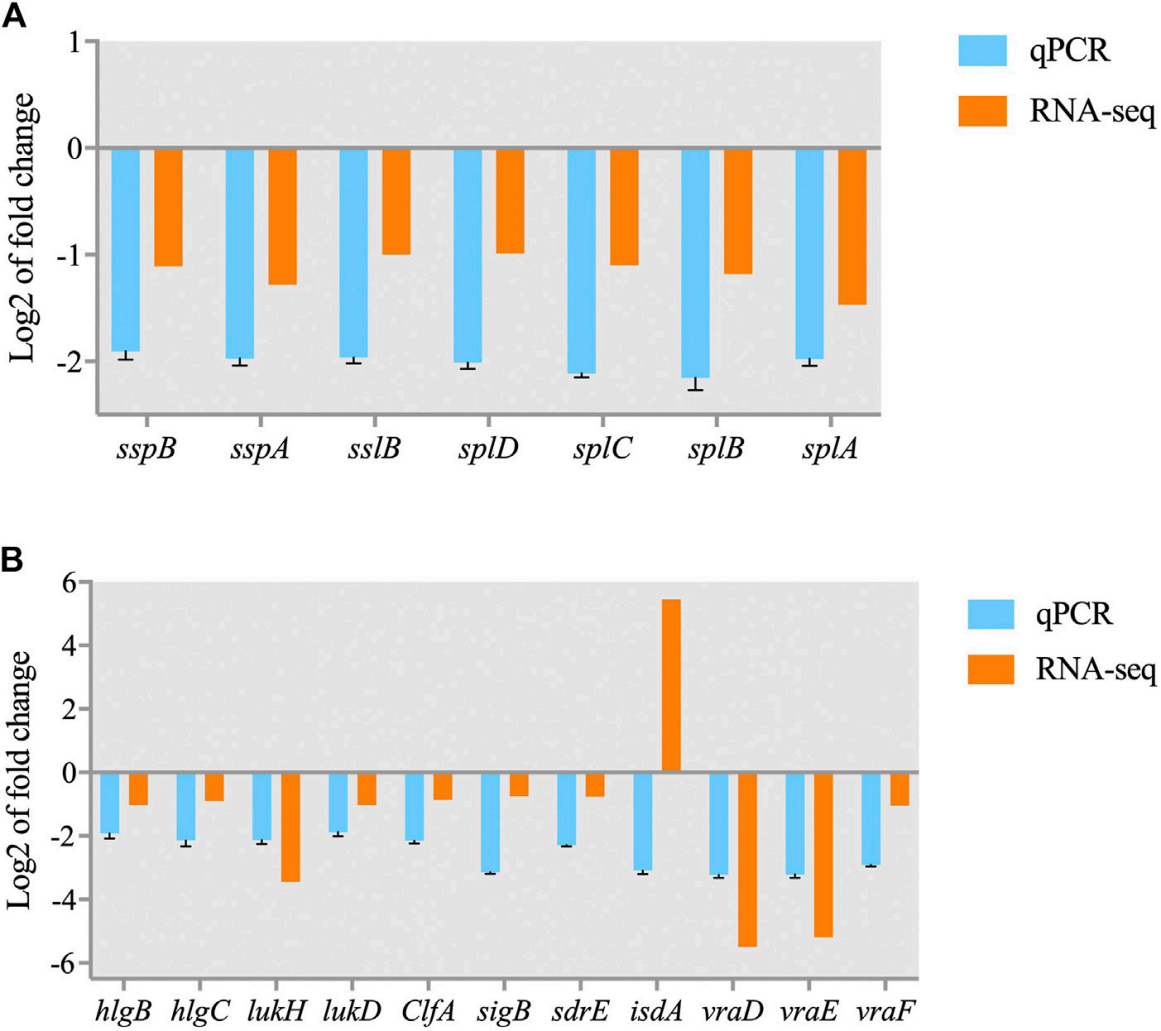
Validation of RNA-seq data for selected genes by real-time PCR. **(A)** DEGs involved in the quorum sensing pathway; **(B)** DEGs involved in *S. aureus* infection.

Some DEGs were selected for qRT-PCR to verify the proteomic and transcriptomic data of previous experiments. As shown in Figure 9, the gene expression patterns demonstrated by the qPCR results were consistent with the transcriptome data with the exception of that of *isdA*. Although the magnitude of the expression FC difference for each gene differed in the qRT-PCR and RNA-seq data, similar expression trends were observed, which suggests that the results of the transcriptomics and proteomics analyses were reliable.

## 4 Discussion

One of the advantages of omics technologies is the capacity to accurately assay large numbers of molecules that may be present at only trace levels; however, this characteristic complicates efforts to analyze the responses of bacteria to drugs or environmental stresses. Therefore, we utilized functional annotation and enrichment analyses to reveal the likely biological and molecular roles of the large number of TA-responsive DEGs and DEPs identified in this study, allowing us to draw conclusions based on our relatively large proteomics and transcriptomics datasets. The functional annotation and enrichment analyses of the DEGs and DEPs identified in *S. aureus* exposed to the 1/2 MIC of TA showed that the DEPs were mainly enriched in ABC transporters, two-component signaling systems, quorum sensing, ribosome, and cell metabolism-related pathways, such as fatty acid biosynthesis, PG biosynthesis, and glycerolipid and glycerophospholipid metabolism, among others.

### 4.1 TA damages the cell membrane envelope

**FIGURE 10:**
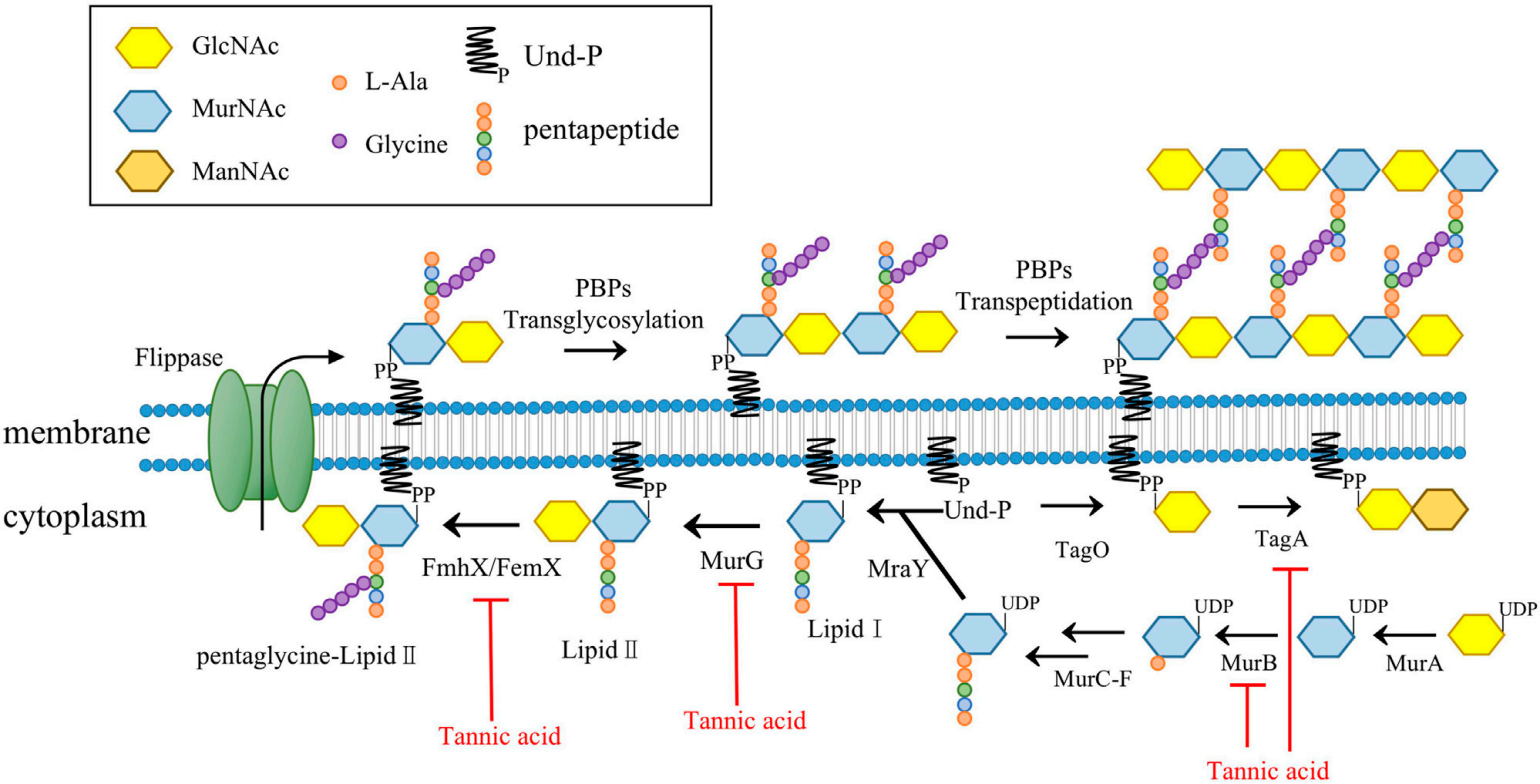
TA target the PG synthesis of *S. aureus*.

GlcNAc, N-acetylglucosamine; MurNAc, N-acetylmuramic acid, ManNAc: N-acetylmannosamine; Und-P, Undcaprenyl phosphate; MurB, UDP-N-acetyl allylacetone glucosamine reductase; MurG, undecaprenyl diphospho-muramoyl pentapeptide beta-N-acetylglucosaminyl transferase; MraY, UDP-MurNAc-pentapeptide phosphotransferase; PBP, penicillin-binding proteins; FmhX/FemX, type 1 glutamine amidotransferase; TagA, glycosyltransferase catalyze the first step of wall teichoic acid synthesis.

As the pathways responsible for producing the main components in the cell wall of Gram-positive bacteria, the synthesis pathways of PGs and teichoic acids each play an important role in cell viability (Gutierrez et al., 2018). The enzymes involved in the PG synthesis pathway are targets of many clinically used antibiotics, such as vancomycin and β-lactam antibiotics (Hua et al., 2019). Previous experiments on methicillin-resistant *S. aureus* (MRSA) suggest that TA might interfere with cell membrane integrity and inhibit the formation of MRSA biofilm, but the mechanism underlying these effects was not reported (Dong et al., 2018). Our microscopy assays showed that direct exposure to TA shrank the outermost cell wall of *S. aureus* and disrupted cell membrane integrity. The expression levels of several proteins related to PG synthesis in *S. aureus* were significantly decreased after TA treatment, including UDP-N-acetylmuramate dehydrogenase (*murB*), type 1 glutamine amidotransferase, peptidoglycan interpeptide bridge formation enzyme (*fmhX/fmhB*) and undecaprenyl diphospho-muramoyl pentapeptide beta-N-acetylglucosaminyl transferase (*murG*) (Table 1). MurB catalyzes the final step in the formation of UDP-N-acetylteichoic acid (UDP-MurNAc), which can act as an essential catalyst in the presence of NADPH to catalyze enolpyruvyl UDP-N-acetylglucosamine to UDP-MurNAc (Winstel et al., 2014). We also found that N-acetylmuramic acid 6-phosphate etherase (murQ), a transcriptional regulator of MurNAc-6p in *S. aureus*, was significantly downregulated at the protein level (Curiel et al., 2011; Gould, 2018). MurG belongs to the glycosyltransferase family and connects the GlcNAc of UDP-GlcNAc to lipid I after MraY catalysis and lipid II production (Hua et al., 2019). MurG and other glycosyltransferases are known to be involved in the synthesis of teichoic acids. In the present study, several glycosyltransferases were significantly downregulated at the protein level in the TA treatment group, including *murG, tagA*, and B4602_RS03245 (Figure 10) (Zhang et al., 2021).

As important components of cell membranes, fatty acids play a very important role in maintaining cell architecture and permeability (Adnan et al., 2017). Three key enzymes involved in fatty acid biosynthesis were significantly downregulated at the protein level in the TA treatment group: acetyl-CoA carboxylase biotin carboxylase subunit (*accC1*), acetyl-CoA carboxylase biotin carboxyl carrier protein subunit (*BCCP*) and *purK*. TA has been shown in previous studies to inhibit b-ketoacyl-ACP reductase (FabG) in the fatty acid synthesis pathway, but the results of this study did not confirm this finding (Wu et al., 2010). In this study, FabG was slightly downregulated at the gene level and showed no significant change at the protein level. In contrast with our findings, others have reported that TA can affect the cell wall integrity of *Lactobacillus plantarum* by increasing the synthesis of three proteins associated with cell wall synthesis (Akanuma et al., 2014). Our finding that TA exposure significantly decreased the protein and/or gene levels of enzymes associated with the PG synthesis and fatty acid synthesis pathways suggests that TA can affect the integrity of the cell envelope.

### 4.2 Ribosome and protein synthesis

Ribosomes perform the important biological function of translating the genetic information of mRNA into proteins. Ribosomes are also crucial targets for many antibiotics, and about 60% of approved antibiotics work by targeting them (Suzuki et al., 2014). Previous transcriptomic studies have shown that tannins purified from *Phyllanthus columnaris* stem bark affect MRSA growth mainly by inhibiting the ribosome pathway, disrupting the translation process and inhibiting protein synthesis (Wilson, 2014). Bacterial ribosomes are composed of the 50s subunit and the 30s subunit; the 50s subunit is composed of 23s and 5s RNA and more than 30 proteins. TA treatment can significantly reduce the protein content or gene expression level of various proteins that compose the *S. aureus* 50S and 30S ribosome subunits, such as 50S ribosomal proteins L34, L36, and L16, among others (Table 1). These ribosomal proteins play an important role in 70s ribosome assembly. The 50s ribosome protein L34 (*rpmH*) is required for efficient 70s ribosome formation. A previous study reported a significant reduction in the binding affinity of 50S ribosome protein L16 (*rplP*) due to a lack of L34 in the ∆rpmH defect strain, leading to conformational changes in the 50S subunit (Li et al., 2015). Another study found that a mutation in ribosomal protein L2 (*rplB*) not only reduced the efficiency of formation of the 70S ribosome, but also induced a deficiency of L16 in the 50S subunit (Usachev et al., 2020). We also found that the protein level of translation initiation factor IF-2 (*infB*), which is involved in regulating the efficiency and fidelity of the formation of translation-initiation complexes, was significantly reduced by TA exposure (Suzuki et al., 2014; Zallot et al., 2017).

Moreover, the protein levels of both ribosome silencing factor (*rsfS*) and ribosome-associated translation inhibitor (*raiA*) were significantly increased by TA treatment. RsfS can inhibit translation by directly binding to the L14 protein on the *Mycobacterium tuberculosis* 50S ribosome to prevent the formation of the 70S initiating complex (Thoden et al., 2004). RaiA can interact with the 70S and 30S ribosome subunits and suppress translation initiation in *Escherichia coli* (Yin et al., 1998). In addition, TA treatment also significantly decreased the expression levels of enzymes associated with tRNA amino acid transport, such as serine-tRNA ligase (*serS*), cys-tRNA (Pro) deacylase (*ybaK*), and epoxyqueuosine reductase (*queH*) (Huo and Viola, 1996). These results indicate that *S. aureus* slow their metabolism and reduce the intensity of protein synthesis in response to TA stress.

### 4.3 Amino acid and purine biosynthesis and metabolism

Amino acids are important raw materials for the synthesis of proteins and nucleotides, so their metabolic pathways are crucial for microbial survival. The formation of carbamoyl phosphate catalyzed by carbamoyl phosphate synthetase yields an important form of inorganic nitrogen that can be utilized by microorganisms and also plays an important role in the biosynthesis of pyrimidine and arginine. Carbamoyl phosphate synthetase and glutamine synthetase (GlnA) are both important enzymes that catalyze the conversion of ammonia into organic nitrogen. The large and small subunits *carB* and *carA,* which constitute carbamoyl phosphate synthetase, were significantly downregulated at the protein and transcript levels (Xu and Grant, 2013). GlnA catalyzes the formation of L-glutamate into L-glutamine, and both of these molecules are important intermediates in the synthesis of tryptophan, histidine and other amino acids (Koenigsknecht et al., 2007). GlnA protein and gene levels were significantly reduced, which indicated that TA treatment affected amino acid and purine synthesis in *S. aureus*. Homoserine kinase (ThrB), which catalyzes the conversion of L-homoserine to L-homoserine phosphate, is an enzyme in the aspartate pathway involved in the synthesis of threonine and isoleucine (Morar et al., 2006). Bifunctional threonine ammonia-lyase/L-serine ammonia-lyase (TdcB) catalyzes L-threonine to form intermediate product 2-oxobutanoate of the L-isoleucine synthesis pathway. The protein and gene levels of *thrB* and *tdcB* were both reduced significantly (Mishima et al., 2001).

The carbon and nitrogen in the purine ring skeleton are mainly provided by aspartic acid, glutamine and glycine, so the synthesis of purine nucleotides is closely related to the anabolism of amino acids. Amidophosphoribosyl transferase (PurF), phosphoribosylformylglycinamidine synthase I (PurQ), adenylosuccinate synthase (B4602_RS00095), phosphoribosylglycinamide formyltransferase (PurN) and adenylosuccinate lyase (PurB) are enzymes involved in the hypoxanthine nucleotide and adenine nucleotide synthesis pathways (Table 1). PurF catalyzes the formation of phosphoribosylamine from phosphoribosylpyrophosphate (PRPP) and glutamine (Cui and Davidson, 2011). PurQ catalyzes the ATP-dependent conversion of formylglycinamide ribonucleotide (FGAR) and glutamine to yield formylglycinamidine ribonucleotide (FGAM) and glutamate (Orelle et al., 2019). The protein and transcript levels of each of the amino acid anabolism-related enzymes mentioned above were decreased significantly by exposure to TA. These results suggest that treatment of *S. aureus* with TA decreased levels of amino acids, especially L-glutamine, threonine, and isoleucine, as well as purine synthesis, inhibiting bacterial growth. Moreover, increased expression of *pruA, lpdA*, and B4602_RS00640, which are involved in valine, leucine, isoleucine and lysine degradation, demonstrated that the amino acid metabolism of *S. aureus* accelerated significantly after TA treatment.

### 4.4 ABC transporter

ABC (ATP-binding cassette) transporters, including importers and exporters, are a class of transmembrane transporters utilized by a wide variety of organisms (Slamti and Lereclus, 2019). ABC importers in bacteria play critical roles in mediating uptake of micronutrients, including saccharides, amino acids, short peptides, phospholipids, cholesterol, and metal ions (Teichmann et al., 2017). In addition, ABC transporters have been shown to protect bacteria from hazardous compounds (Nguyen and Gotz, 2016). In the present study, we found that ABC transporter proteins associated with iron transport and amino acid uptake were significantly affected by TA treatment (Table 1). Oligopeptide permeases consist of five protein components. The transport process, which delivers extracellular peptides to membrane components, depends on ATP hydrolysis by two intracellular ATPase subunits, OppD and OppF (Koster, 2001). Our analyses revealed upregulation of OppD, OppF and branched-chain amino acid transporter (B4602_RS11480) proteins, and downregulation of HisM and GmpC proteins, which indicated that amino acid transportation was disrupted by TA. Bacteria accumulate stress-relieving compounds such as betaine, choline, and carnitine *via* ABC transporters in response to increased external osmolarity by balancing the osmotic gradient across the cytoplasmic membrane (Grigg et al., 2010). *opuBB, opuCC* and *opuBA*, which encode the extracellular substrate-binding region of the ABC-type glycine betaine transport system (Lawrence et al., 1998), were significantly downregulated at the protein level after TA treatment. These findings suggest that TA may affect osmotic pressure by inhibiting the glycine betaine transport system.

Iron, which is a key molecule involved in cytochrome formation, resistance to reactive oxygen species and other molecular functions, is an essential nutrient for most living bacteria. *S. aureus* expresses a set of transport proteins that are devoted to the acquisition of several forms of iron (e.g., Fe^2+^, Fe^3+^-siderophore, and heme) from the environment (Durgadevi et al., 2020). We found that SirA, a lipoprotein in staphyloferrin B with high affinity and high specificity (Rapun-Araiz et al., 2020), was significantly upregulated. The transporters known to mediate uptake of iron (*isdF, fepC*), ferric citrate (*fecB, fecCD*) and other inorganic ions were significantly elevated at the protein and transcript levels, with the exception of *psaA* (Table 1)*. PsaA* is likely to be a Mn^2+^ binding protein and has been identified as an essential virulence factor in a mouse model (Bleul et al., 2021). Our results are consistent with a previous report showing that TA upregulated heme- and iron-related ABC transporter proteins in *Proteus mirabilis* (Wilde et al., 2015). Previous studies have suggested that TA may chelate iron from the medium to render it unavailable to microorganisms (Chung et al., 1998b). *S. aureus* probably meet their iron needs by up-regulating transporters involved in iron uptake, and these transporters, which are also involved in sugar transport (*ugpA, ugpC*), phosphonate transport (*phnD, phnE*), the lipoprotein export system (*lolD*), and cell envelope biogenesis, were significantly downregulated after TA treatment (Table 1). Taken together, our results suggest that the primary bacteriostatic mechanism of TA is disruption of transport systems related to iron and amino acid uptake, which impairs protein synthesis and destroys cell membranes.

### 4.5 Two-component system

Two-component signal transduction systems (TCS), which are widely found in prokaryotic bacteria, are the main mechanism utilized by bacteria to regulate metabolism in response to environmental changes or cope with stress factors such as antibiotics (Qiu et al., 2021). *S. aureus* encodes 16 TCSs to sense and adapt to environmental changes primarily by regulating the expression of virulence genes (Pietiainen et al., 2009). The staphylococcal respiratory response (SrrAB) system regulates the expression of genes involved in anaerobic metabolism, nitrosative stress, and cytochrome biosynthesis (Sass et al., 2008). TA treatment significantly reduced the protein levels of SrrA and SrrB in *S. aureus*, but their gene expression levels were not significantly reduced, suggesting that TA may impair cytochrome biosynthesis and decrease resistance to nitrosative stress (Popella et al., 2016) (Table 1).

TA treatment has been shown to inhibit some TCS elements associated with bacterial resistance to antibiotics at the protein and transcript levels. For example, the two-component ABC transporter VraDE is involved in the regulation of antimicrobial peptide resistance against bacitracin and human beta-defensin 3 as a downstream signal of the GraRS system (Yang et al., 2012; Xu et al., 2017; Li et al., 2019). The activation of GraRS also induces expression of the resistance factor protein DltABDCX, which contributed to the net positive surface charge by covalently incorporating D-alanine into cell wall teichoic acids (Hua et al., 2018). In this study, TA significantly decreased the protein and mRNA levels of *vraDE* and *vraF*, as well as the mRNA content of *dltABDC*, in *S. aureus* (1.56-2.32 log2(FC), data not shown), indicating that the cell envelope was damaged. TA can also significantly inhibit the transcription and translation of *uhpT* and *glpT*, which encode the glucose 6-phosphate transporter and glycerol-3-phosphate transporter of *S. aureus*, to enhance fosfomycin resistance (Wu et al., 2019).

## 5 Conclusion

In this study, transcriptome and proteome data were combined to discover the antibacterial mechanism of TA against *S. aureus* for the first time. The antibacterial mechanism of TA was mainly attributed to destruction of the cell envelope as a result of its inhibitory effects on the production of PGs, teichoic acids and lipids, as well as inhibition of ribosome formation and disruption of protein synthesis and amino acid biosynthesis. We speculate that the main mechanism of TA is depression of cell envelope synthesis and ribosome formation, and related mechanisms will be further explored in future studies.

## Data Availability

The datasets presented in this study can be found in online repositories. The names of the repository/repositories and accession number(s) can be found below: NCBI BioProject [https://www.ncbi.nlm.nih.gov/bioproject/], PRJNA957484; ProteomeXchange [https://proteomecentral.proteomexchange.org/cgi/GetDataset], PXD041839.

## Supplementary material

SUPPLEMENTARY TABLE S1 The primer sequences used in qRT-PCR.
