# Supplementary material for: Combined proteomic and transcriptomic analysis of the antimicrobial mechanism of tannic acid against Staphylococcus aureus
Source: Front Pharmacol. 2023 Aug 16;14:1178177. doi: 10.3389/fphar.2023.1178177 (PMC10466393; doi:10.3389/fphar.2023.1178177)
Supplement: Supplementary file 1 [file DataSheet1.docx]

**Table S1.** The primer sequences used in qRT-PCR

| Genes | Sequence（5’-3’) | Product size |
| --- | --- | --- |
| *sspB*-F | AGCCAAAGCCGATTCACACT | 100 |
| *sspB*-R | ACCAGCAAATTGTTGTTGTGCT |  |
| *sspA*-F | AACAGCGACACTTGTGAGTTCTCC | 85 |
| *sspA*-R | GCTTGACTGCGTTTGTTGTGGATG |  |
| *sslB*-F | GGTTTAGATGTCTTTGCGGTACCA | 118 |
| *sslB*-R | AGTTCGGCGTTCTTAGAGACTCA |  |
| *splD*-F | ATGGTATTCAACAAACGGCCAAAGC | 93 |
| *splD*-R | CGCCCATCCATGTAACACCACTG |  |
| *splC*-F | ATTACTGCCCATCCAAACGGTGAC | 127 |
| *splC*-R | GTCCACGTTCAACTGCTTGTTCTTC |  |
| *splB*-F | TGGGCGATAGAATTACTGCACATCC | 127 |
| *splB*-R | TCCACGTTCTATTGCACGCTCTTC |  |
| *splA*-F | CGGGTGGTACTGGTGTAGTTGTTG | 135 |
| *splA*-R | CGTCGTAGTTTCCTCCGCCTTTAC |  |
| *lukH*-F | AGCAACGACTCAAGCAAATTCAGC | 141 |
| *lukH*-R | TTCCCAATATCATCCGGTGCTGTTG |  |
| *lukD*-F | GGCGCGTGGTAACTTTAACCC | 142 |
| *lukD*-R | ACCAATCCAGTGTAGTCGATTCCA |  |
| *hlgC*-F | AAAGTGTTTTATGGGGCGTCAAAGC | 147 |
| *hlgC*-R | GAGGTGGTAACTCACTGTCTGGAAC |  |
| *hlgB*-F | AATGTTGGCTGGGGAGTTGAAGC | 118 |
| *hlgB*-R | TGCTTTGTCTGCCAGCTAAGAAGAG |  |
| *ClfA*-F | CAGCGAGCGATTCAGACTCAGAC | 109 |
| *ClfA*-R | GAGTCGGAATCACTGTCGGAATCTG |  |
| *sdrE*-F | TCAGACTCAGACAGCGACTCAGAC | 81 |
| *sdrE*-R | TGTATGTTTTCCTGCATCCGAGTCC |  |
| *sigB*-F | GTCCTTTGAACGGAAGTTTGAAGCC | 102 |
| *sigB*-R | CGTCTCGGAACATGTACACTCCAAG |  |
| *isdA*-F | GCAACAGAAGCTACGAACGCAAC | 112 |
| *isdA*-R | TGTGTGACTTCTCTGAAGAGCCATC |  |
| *vraD*-F | TACACATGATCCGGTTGCAGCAAG | 103 |
| *vraD*-R | GGCCTGTTTAGAACGTCCTTCCTG |  |
| *vraE*-F | GGTGTATTATGTGGCATTGCAGGTG | 104 |
| *vraE*-R | AGGGCCATAGGTTCGAAATGTATCG |  |
| *vraF*-F | GGCACAAGAAGTGTTGCGAGATATC | 93 |
| *vraF*-R | AATGTCGTTTTCCCAGATCCAGAGG |  |
